# Developing the Polish Educational Needs Assessment Tool (Pol-ENAT) in rheumatoid arthritis and systemic sclerosis: a cross-cultural validation study using Rasch analysis

**DOI:** 10.1007/s11136-014-0805-6

**Published:** 2014-09-18

**Authors:** Matylda Sierakowska, Stanisław Sierakowski, Justyna Sierakowska, Mike Horton, Mwidimi Ndosi

**Affiliations:** 1 Department of Integrated Medical Care, Medical University of Bialystok, Białystok, Poland; 2 Department of Rheumatology and Internal Diseases, Medical University of Bialystok, Białystok, Poland; 3 Department of English Literature, University of Bialystok, Białystok, Poland; 4 Academic Department of Rehabilitation Medicine, Leeds Institute of Rheumatic and Musculoskeletal Medicine, University of Leeds, Leeds, UK; 5 School of Healthcare, University of Leeds, Leeds, LS2 9JT UK

**Keywords:** Rheumatoid arthritis, Systemic sclerosis, Patient education, Needs assessment, Cross-cultural validation, Rasch analysis

## Abstract

**Objectives:**

To undertake cross-cultural adaptation and validation of the educational needs assessment tool (ENAT) for use with people with rheumatoid arthritis (RA) and systemic sclerosis (SSc) in Poland.

**Methods:**

The study involved two main phases: (1) cross-cultural adaptation of the ENAT from English into Polish and (2) Cross-cultural validation of Polish Educational Needs Assessment Tool (Pol-ENAT). The first phase followed an established process of cross-cultural adaptation of self-report measures. The second phase involved completion of the Pol-ENAT by patients and subjecting the data to Rasch analysis to assess the construct validity, unidimensionality, internal consistency and cross-cultural invariance.

**Results:**

An adequate conceptual equivalence was achieved following the adaptation process. The dataset for validation comprised a total of 278 patients, 237 (85.3 %) of which were female. In each disease group (145, RA and 133, SSc), the 7 domains of the Pol-ENAT were found to fit the Rasch model, *X* ^2^(*df*) = 16.953(14), *p* = 0.259 and 8.132(14), *p* = 0.882 for RA and SSc, respectively. Internal consistency of the Pol-ENAT was high (patient separation index = 0.85 and 0.89 for SSc and RA, respectively), and unidimensionality was confirmed. Cross-cultural differential item functioning (DIF) was detected in some subscales, and DIF-adjusted conversion tables were calibrated to enable cross-cultural comparison of data between Poland and the UK.

**Conclusion:**

Using a standard process in cross-cultural adaptation, conceptual equivalence was achieved between the original (UK) ENAT and the adapted Pol-ENAT. Fit to the Rasch model, confirmed that the construct validity, unidimensionality and internal consistency of the ENAT have been preserved.

**Electronic supplementary material:**

The online version of this article (doi:10.1007/s11136-014-0805-6) contains supplementary material, which is available to authorized users.

## Introduction

Rheumatoid arthritis (RA) is a systemic inflammatory disease characterized by the presence of a destructive polyarthritis with a predisposition for affecting the peripheral joints [1]. Systemic sclerosis (SSc) is an autoimmune connective tissue disease characterized by small-vessel vasculopathy, autoantibody production and excessive collagen deposition in the skin and internal organs [2]. Both RA and SSc are chronic disabling diseases, which have a negative impact on individuals’ physical, social and psychological functioning [3–5].

People with RA and SSc have many and varied needs, and so in addition to offering specific drug treatments and therapies, patient education is recommended as an integral part of the disease management [6]. Most patients with rheumatic diseases believe in the value of patient education and health professionals; especially, nurses spend a considerable amount of their time in providing patient education [7–9].

Patient education is an interactive process between patients and health professionals aimed at supporting and enabling patients to manage their life with arthritis and optimizing their health and well-being [10]. The effects of patient education can be difficult to demonstrate in randomized controlled trials although there is growing evidence that patients’ needs and individual learning capabilities play an important role [9–12]. In chronic diseases, patients’ perspective of their educational needs is important since they have experiential knowledge about their disease and they undertake daily self-care activities. Patients’ expectations determine whether patient education is likely to lead to behavioural change [13]; therefore, it is extremely important for health professionals to assess patient’s educational needs and priorities before providing education. This assessment will help tailor education to meet patient’s priorities and promote shared decision-making.

Although it is widely accepted that effective patient education has to be targeted to meet the patient’s needs and expectations [14–16], our literature search found only one tool for assessing educational needs of patients with rheumatic diseases: the educational needs assessment tool—ENAT [17], which was developed in the UK. The tool has been adapted into other eight European languages (Austrian German, Dutch, Italian, Finnish, Norwegian, Portuguese, Spanish and Swedish) and validated in RA, SSc and other rheumatic conditions [18, 19], but there was no Polish version of the questionnaire.

For a questionnaire to be used in two different cultures, it must be shown to demonstrate conceptual and measurement equivalence. C*onceptual equivalence* can be defined as similarities in the way abstract, latent concepts are interpreted among different cultural groups [20]. To achieve this in the questionnaires adaptation, different guidelines have been proposed, most of which involve a rigorous iterative ‘forward–backward’ translation process, review of the translations and testing on a sample of patients [21–24]. Measurement equivalence is the comparability of psychometric properties in the source and the target (adapted) measures [25]. Questionnaire items do not always function equally in different cultural groups, and an item that behaves differently is said to exhibit a cross-cultural bias or differential item function (DIF) with respect to culture [26–28]. Since measurement equivalence is the basic requirement for comparing data across cultural groups, it is important to: (1) assess the construct validity of the adapted questionnaire and (2) examine and account for cross-cultural bias in the translated questionnaire [26, 28]. Rasch analysis is the method by which both construct validity and cross-cultural DIF can be assessed [27–29]. The aim of this study was to undertake a cross-cultural adaptation of the ENAT into Polish and then use Rasch analysis to validate the adapted tool (Pol-ENAT) in RA and Ssc.

## Methods

### Design

This was a cross-sectional study conducted in two phases: (1) cross-cultural adaptation of the ENAT into Polish and (2) validation of the adapted tools (Pol-ENAT) in RA and SSc. The first (adaptation) phase followed standardized guidelines for cross-cultural adaptation of patient-reported outcome measures suggested by Beaton et al. [21]. The second phase was conducted using a cross-sectional survey design requiring patient completion of the adapted versions of the ENAT on one occasion, then subjecting the data to Rasch analysis to assess the construct validity, reliability and cross-cultural invariance of the translated tools. Ethical approval was obtained from the local ethics committee.

### Measures

The ENAT is a simple patient-completed questionnaire, which comprises 39 items grouped into the following 7 domains: managing pain (6 items), movement (5 items), feelings (4 items), arthritis process (7 items), treatments (7 items), self-help measures (6 items) and support systems (4 items). Items are Likert scales ranging from 0—“not important at all” to 4—“extremely important”. The ENAT is used as a ‘generic’ tool across rheumatic diseases but the term ‘arthritis’ is replaced by ‘rheumatic disease’ when used in SSc. The descriptions of how the ENAT is used and scored are given in the online supplementary material.

### Phase 1: Cross-cultural adaptation

The original (English) ENAT was translated into Polish using the cross-cultural adaptation process described by Beaton et al. [21]. The process comprises five stages: Forward translation, synthesis of the translations, back-translation, expert committee assessment and field testing.

The first (forward) translation stage from English (source language) into Polish (target language) was carried out by two independent translators whose mother tongue was Polish. The first translator was a professional bilingual translator (SS), and the second was a bilingual lay person (TS). Each translator produced a written report, (T1 and T2) of his translation, highlighting difficult phrases or uncertainties along with the rationale for their word choices.

A third unbiased person was added to the team (JS), whose role was to serve as a mediator in the discussion of translation differences arising from T1 and T2. Working from the original questionnaire as well as from the first (T1) and the second translator’s (T2) version, one common translation (T-12) was produced together with a report documenting the process and how issues were resolved.

Back-translation stage was undertaken by two bilingual ‘back-translators’ (MK and PD) whose mother tongue was English and totally blind to the original versions. They worked from the T-12 version of the ENAT, producing English translations (BT1 and BT2). This is a process of validity checking to ensure the translated version accurately reflects the item content of the original version.

The composition of the expert committee included a methodologist, health professionals, all the (forward and backward) translators and the translation synthesis recorder. The original developer of the questionnaire was also included. The expert committee consolidated all the versions and components of the questionnaire and all translated versions (T1, T2, T12, BT1, BT2), discussing discrepancies raised in previous stages, and a consensus was reached on all items. The prefinal version of the ENAT was produced for field testing.

The field test of the adapted ENAT comprised 30 patients (15 with RA and 15 with SSc) recruited from the rheumatology outpatient clinics at one of the recruiting centres. They completed the Pol-ENAT unaided; then, they were interviewed to probe what they thought was meant by each questionnaire item and their response. Both the meaning of the items and responses were explored. A summary of issues raised in the translation for each item is presented in Table 2.

### Phase 2: Cross-cultural validation

The final translated versions of the ENAT (for RA and SSc) were then completed by a consecutive sample of patients fulfilling the above-mentioned inclusion criteria. The ENATs were anonymous but contained patients’ demographical data such as gender, age, educational background and self-reported disease duration. Once completed, the data were entered into an IBM SPSS database (version 19) [30] and subsequently subjected to Rasch analysis RUMM2030 software [31].

### Participants

Patients were recruited from rheumatology outpatient clinics at seven rheumatology centres in Poland. The inclusion criteria were: (1) positive diagnosis of either RA (using the ACR and the ACR/EULAR classification criteria for [32, 33]) or SSc (2) aged 18 years or above and (3) willingness to complete and return a questionnaire. The exclusion criteria were: (1) inability to complete the ENAT unaided and (2) having more than one form of rheumatic disease. The patients in the SSc group had articular symptoms such as pain, stiffness and movement impairment. Matching datasets of patients with RA and SSc from the UK were used in the analysis stage to test the cross-cultural invariance. This would inform whether the ENAT works in the same manner when completed by patients with the same disease in English or in Polish (the adapted version).

### Data analysis

Since the original (English) ENAT and other language versions have been shown to fit the Rasch model after correction for local dependency [18, 19], we used Rasch analysis in this study to test whether Pol-ENAT had retained its psychometric properties following the adaptation process. For this analysis, we used the Master’s Partial Credit Model parameterization [34] in RUM2030 software [31]. Each Pol-ENAT item was assessed for ‘fit’ to the Rasch model, and then, the scale was assessed as a 39-item construct and as a 7-domain construct. In addition to fit statistics, the scales were tested for internal consistency, unidimensionality and DIF. The expected values for perfect model fit are presented at the bottom of tables of results. DIF analysis can be carried out efficiently along with other psychometric tests within the framework of Rasch models. Rasch analysis was used to test the evidence of cross-cultural DIF, to quantify the DIF and to calibrate a DIF-adjusted interval-level scale [27, 35]. Where cross-cultural DIF (between the Polish and UK datasets) was found, a DIF-adjusted scale was calibrated for use when data pooling from the two countries is desired.

## Results

### Patient characteristics

The validation study sample comprised 278 patients with RA (*n* = 145) and SSc (*n* = 133). Their mean (SD) age, disease duration and gender distribution are summarized in Table 1. The cross-cultural validation phase included comparison of datasets of UK patients with RA and SSc, and their patient characteristics are also summarized in Table 1.

**Table 1:** Patient characteristics

	Poland		UK	
	RA (*n* = 145)	SSc (*n* = 133)	RA (*n* = 125)	SSc (*n* = 128)
Mean age (SD)	52.79 (13.05)	53.89 (14.26)	56.26 (13.24)	54.44 (12.07)
Disease duration (SD)	13.36 (9.84)	10.84 (10.34)	13.55 (9.52)	14.34 (11.49)
% Female	82.1	88.7	79.2	79.7
% Only basic education	9.7	10.5		

*SD* standard deviation, *RA* rheumatoid arthritis, *SSc* systemic sclerosis

### Adaptation into Polish

Issues around translation included: multiple meanings of certain concepts, grammatical difficulties and inexactness or idiomatic expressions. Other issues were due to differences in the style of formulating questionnaire items in English and in Polish. The translators and the expert committee solved all the above problems by finding Polish equivalents, which would be understandable but also accurate from a medical point of view. A summary of issues arising from the back-translations and the agreements for each ENAT item (for RA) are presented in Table 2. The Expert Committee believes that the aim of proposing an accurate Polish version of the ENAT has been achieved.

**Table 2:** Back-translation, issues and agreements for each ENAT item

Original	Back-translation 1 (BT1)	Back-translation 2 (BT2)	Issues	Agreement
Arthritis Educational Needs Assessment Tool	“Assessment Tools for Educational Needs of Patients with Arthritis”	“A tool for assessing educational needs of patients with arthritis related”	Uncertainty word whether “tools” or “tool” should in be used in Polish version Discussion on the Polish multiple meaning of word “arthritis” Also, there were differences in translation of the term “arthritis”	“Narzędzie do oceny potrzeb edukacyjnych pacjentów związanych z zapaleniem stawów” The chosen term “zapalenie stawów” is the most adequate translation.
How long have you had your arthritis for?	“How long have you had arthritis?”	“How long have you suffered from arthritis?”	Uncertainty whether word “suffer” is appropriate	“Jak długo choruje Pani/Pan na zapalenie stawów ?” The Polish translation is correct in terms of style.
Please state your age in years:	“Please state your age”	“Please state your age”	Discussion whether phrase “in years” should be added	“Proszę podać swój wiek.” The phrase “in years” has been omitted.
How old were you when you left school?	“How old were you when you finished with formal education?”	“How old were you when you finished school education?”	Customary question concerning education in Polish does not require information about age	“Proszę podać swoje wykształcenie.” The most correct Polish phrase has been chosen.
At this time, do you want education about anything to help you deal with your arthritis?	Would you currently like to receive information which will help you manage your arthritis?	Are you interested in getting information that will help you cope with arthritis?	Uncertainty how to translate into Polish “manage”, “cope with” and “education”	“Czy obecnie chciałaby Pani/Pan zdobyć informacje które pomogą Pani/Panu radzić sobie z zapaleniem stawów?” Polish translation is correct in terms of style and grammar.
If yes, what?	“If yes, what information would you like to receive?”	“If so, what would you like to know?”	More formally correct version of question in Polish should be given	“Jeśli tak, to czego chciałaby Pani/Pan się dowiedzieć?” Polish version is correct in terms of style.
In general, how much information do you want about your arthritis?	“In general, how much would you like to know about your rheumatic disease?”	“Generally speaking, how much would you like to know about your rheumatism?”	Discussion on word choice between “rheumatic disease” and “rheumatism”	“Ogólnie rzecz biorąc, jak dużo chciałaby się Pani/Pan dowiedzieć o swojej chorobie reumatycznej?” Polish version applies the term “rheumathic disease”
How much do you need to know now about each of the following things? Please tick in the column that shows best how you feel:	“How much would you like to know right now about the following issues? Please mark the appropriate column with an ‘X’”	“How much would you/you already know about the following issues? Please place a cross in the appropriate column”	Discussion concerning the phrase “please place a cross in the appropriate column”	“Jak wiele chciałaby Pani/Pan wiedzieć już teraz na temat następujących zagadnień? Proszę o postawienie krzyżyka w odpowiedniej kolumnie.” The most adequate Polish equivalent has been chosen.
This section relates to managing pain	“Section on managing pain”	“The section on coping with pain”	Discussion whether to choose less or more formal way of introducing a new section of questions	“Sekcja dotycząca radzenie sobie z bólem.” A more formal way of introducing a new section has been chosen.
How important is it for you to know more about the following:	How important is knowing more about the following issues for you?	How important is it for you/you would know more about the following issues?	Discussion wether choose less or more formal way of asking a question	“Jak ważne jest dla Pani/Pana by wiedzieć więcej o następujących zagadnieniach.” A formal way of asking a question has been chosen.
Using exercise	“Gymnastic exercise”	“Physical exercises”	Uncertainty whether “gymnastic” and “physical” can be used synonymic	“Ćwiczenia gimnastyczne” Polish version is correct in terms of style.
This section relates to movement	“Section on issues related to moving around”	“The section on issues related to mobility”	Discussion whether to choose less or more formal way of introducing a new section of questions	“Sekcja dotycząca zagadnień związanych z poruszaniem się.” The most adequate Polish version has been chosen
Ways to do things which wear my joints less	“Methods of reducing wear of/relieving joints”	“Methods of relieving joints”	English idiomatic expression “wear joints less” was replaced with a Polish idiomatic phrase “oszczędzać satwy” (In English: save)	“Metody oszczędzania/odciążania stawów”
Ways to deal with moods or depression	“Methods to help manage mood changes or depressive states”	“Ways to cope with moods or periods of depression”	Ambiguous meaning of a term “moods”	“Sponsor radzenia sobie ze zmiennością nastrojów lub stanami depresji.” The chosen term „zmienność nastrojów” means frequent changes in mood
Why I am feeling down or depressed	“Why do I feel down or depressed?”	“Why do I feel moody or depresses?”	Ambiguous meaning of the term “be depressed”	“Dlaczego czują się przygnębiona/y, czy depresyjna/y” The chosen term „depresyjny” relates to a state of depression.
How arthritis might affect my children or relatives	“Can the disease have an effect on the lives of my children and close ones?”	“Can the disease affect the lives of my children and family?”	Multiple meaning of word “affect”	“Czy choroba może mieć wpływ na życie moich dzieci i bliskich?” The chosen phrase „wpływ na życie” relates to quality of life
What might happen in the future	“How will my condition change in the future?”	“How will my condition change in the future?”	The question is open to various interpretations	“Jak mój stan będzie się zmieniał w przyszłości?” The translation focuses on patient’s personal condition in the future
This section is about treatments you may be receiving from health professionals	“Section on methods of treatment that you can receive from nurses and other medical professionals”	“The section concerns treatments that the patient can receive from nurses and other health care workers”	Lack of a Polish equivalent of a term “health professionals”	“Secant dotycząca sposobów leczenia, które Pani/Pan może otrzymać od pielęgniarki i innych pracowników ochrony zdrowia.” The chosen phrase describes the meaning of “health professionals”
How an operation might help me	“Can surgery help me?”	“Can surgery help me?”	Uncertainty whether word “surgery” or “operation” should be used	“Czy zabieg chirurgiczny może mi pomóc?” The most adequate Polish term has been chosen.
What the side effects of my medicines are	“What are adverse side effects of medicines?”	“Are there side effects to the medication?”	Uncertainty whether the question should be more general or relate to condition of the particular patient	“Jakie są działanie niepożądane leków które przyjmuję?” The chosen phrase relates to patient’s condition.
How appliances might help me (splints, adaptations, collars)	“What devices may help me (orthopaedic collars, splints, stabilizers)?”	“What appliances can help me (e.g. orthopaedic collars, splints, stabilizers)?”	Difficulty with translating term “adaptations”	“Jakie przyrządy i/lub udogodnienia mogą mi pomóc (np. kołnierze ortopedyczne, szyny, specjalnie dostosowane poręcze, etc.)?” A descriptive phrase explaining the term “adaptation” has been chosen
Foods or vitamins that might help	“Diet or vitamins which may help”	“Diet or vitamins that may help”	Uncertainty whether “foods” relates to “diet”	“Produkty pokarmowe lub witaminy które mogą pomóc” The chosen term “produkty pokarmowe” means foods
Exercises I should be doing	“Recommended motion exercises”	“Recommended physical exercises”	Uncertainty whether word “recommended” should be used	“Zalecane ćwiczenia” The most adequate Polish translation has been chosen
How much exercise I should be doing	“Frequency of performing motion exercises”	“The frequency of physical exercises”	Uncertainty whether the word “frequency” is adequate	“Częstotliwość wykonywania ćwiczeń ruchowych” The chosen term “częstotliwość” means frequency
Times when I should call the doctor or nurse	“Situations when I should contact a doctor or nurse”	“In what situations, should I consult a doctor or nurse?”	Uncertainty whether “situations” is the correct translation of the word “times”	“Sytuacje w których powinnam/nienem skontaktować się z lekarzem lub pielęgniarką?” Polish translation is correct in terms of grammar and style
Organizations I can get in touch with about arthritis	“Organizations which can help patients with rheumatic diseases?”	“Organizations that can help patients with rheumatic diseases”	Ambiguous meaning of term “organization” also there is an ambiguous meaning of idiomatic expression “get in touch with.”	“Stowarzyszenia które mogą pomóc pacjentom z chorobami reumatycznymi.” Polish phrase describing contacting an organist has been chosen
Who I can ask about financial help	“Who can I ask for financial help?”	“Who can I ask about financial assistance?”	Lack of cultural equivalence (it is not possible to ask for financial help in case of suffering from arthritis)	“Kogo mogę poprosić o pomoc finansową?”
Where I can find groups who will help me to cope with arthritis	“Where can I find support groups for people with rheumatic diseases?”	“Where can I find support groups for people with rheumatic diseases?”	Uncertainty whether “groups” means the same as “support groups”	“Gdzie mogę znaleźć grupy wsparcia dla osób z chorobami reumatycznymi ?” The term related to help groups has been used
How I can get the most out of seeing the doctor or nurse	“How can I improve communication with doctors or nurses to maximize effectiveness?”	“How to make more effective contacts with a doctor or nurse?”	Idiomatic expression “get the most out of” can be translated in various ways in Polish	“Jakeeeeeee sprawić by kontakty z lekarzem lub pielęgniarką były najbardziej efektywne?” The word “efektywne” (in English: effective) has been used

### Cross-cultural validation phase

Table 3 presents fit statistics for individual items and for each subscale, i.e. pain, movement and feelings. A significant chi-square probability suggests a departure from the model. Most items in both the RA and SSc datasets were found to fit the model.

**Table 3:** Fit statistics for individual items and subscales (testlets)

Items		RA						SSc					
		Loc	SE	FR	DF	χ^2^	*P	Loc	SE	FR	DF	χ^2^	*P
Pain	1	−1.09	0.12	0.45	130.49	0.13	0.94	−0.84	0.11	−0.47	122.69	1.43	0.49
	2	−0.01	0.10	1.15	130.49	0.79	0.67	−0.13	0.09	0.64	122.69	1.04	0.59
	3	−0.20	0.10	0.80	130.49	3.66	0.16	0.10	0.09	−0.07	122.69	4.11	0.13
	4	0.20	0.10	1.66	130.49	5.19	0.07	−0.03	0.09	1.00	122.69	0.64	0.73
	5	−0.17	0.10	0.44	130.49	1.21	0.54	−0.21	0.09	1.64	122.69	7.99	0.02
	6	0.13	0.09	3.58	130.49	4.27	0.12	0.02	0.09	2.71	122.69	12.27	**0.00**
Movement	7	0.71	0.10	4.86	130.49	30.85	**0.00**	0.65	0.08	2.13	122.69	2.81	0.25
	8	0.76	0.10	1.71	130.49	8.48	0.01	0.57	0.08	1.09	122.69	1.41	0.50
	9	0.22	0.10	2.47	130.49	6.17	0.05	0.49	0.09	0.92	122.69	0.08	0.96
	10	0.16	0.10	0.97	130.49	1.29	0.52	0.15	0.09	−0.18	122.69	0.40	0.82
	11	−0.76	0.12	−1.02	130.49	2.86	0.24	−0.08	0.08	−0.28	122.69	0.04	0.98
Feelings	12	−0.06	0.10	−0.33	130.49	2.86	0.24	0.09	0.09	0.13	122.69	0.65	0.72
	13	0.31	0.10	0.03	130.49	1.87	0.39	0.17	0.08	1.80	122.69	0.61	0.74
	14	0.10	0.10	−0.92	130.49	0.59	0.74	0.09	0.09	0.11	122.69	0.68	0.71
	15	0.41	0.09	0.59	130.49	0.93	0.63	0.36	0.08	1.66	122.69	0.32	0.85
Disease	16	−0.27	0.09	3.91	130.49	8.56	0.01	−0.33	0.09	1.68	122.69	2.42	0.30
	17	0.18	0.09	0.07	130.49	0.11	0.95	0.17	0.08	0.59	122.69	0.64	0.73
	18	−0.32	0.09	−0.02	130.49	3.01	0.22	−0.32	0.08	1.54	122.69	0.15	0.93
	19	−0.90	0.11	−0.82	130.49	1.36	0.51	−0.28	0.08	0.67	122.69	0.56	0.75
	20	−0.27	0.10	−2.07	130.49	2.37	0.31	−0.11	0.08	−1.10	122.69	2.63	0.27
	21	0.21	0.09	0.62	130.49	0.64	0.73	0.00	0.09	−1.45	122.69	6.23	0.04
	22	−0.59	0.10	−0.19	130.49	0.38	0.83	−0.58	0.09	−0.53	122.69	1.83	0.40
Treatments	23	0.27	0.09	−0.57	130.49	4.65	0.10	0.09	0.09	0.81	122.69	0.93	0.63
	24	−0.01	0.09	−0.39	130.49	1.95	0.38	0.06	0.08	−0.08	122.69	0.74	0.69
	25	−0.42	0.09	−2.26	130.49	6.29	0.04	−0.21	0.09	0.93	122.69	2.26	0.32
	26	0.22	0.09	−1.76	130.49	1.84	0.40	0.05	0.09	−0.17	122.69	0.42	0.81
	27	0.17	0.09	−1.77	130.49	1.18	0.56	−0.01	0.09	−0.53	122.69	1.83	0.40
	28	0.11	0.09	1.67	130.49	0.40	0.82	0.45	0.08	0.50	122.69	3.43	0.18
	29	0.21	0.09	3.10	130.49	2.10	0.35	0.55	0.08	−0.04	122.69	0.25	0.88
Self-help	30	0.32	0.10	1.80	130.49	1.59	0.45	0.19	0.09	3.02	122.69	9.87	0.01
	31	−0.28	0.10	−1.09	130.49	0.92	0.63	−0.33	0.10	0.48	122.69	0.16	0.92
	32	−0.25	0.10	−2.06	130.49	7.26	0.03	−0.46	0.10	−1.18	122.69	6.68	0.04
	33	−0.42	0.10	−1.93	130.49	8.24	0.02	−0.34	0.10	−1.17	122.69	6.02	0.05
	34	−0.19	0.10	−2.30	130.49	8.88	0.01	−0.24	0.10	−0.89	122.69	8.40	0.02
	35	0.05	0.09	−2.23	130.49	2.50	0.29	−0.27	0.09	−2.01	122.69	9.19	0.01
Support	36	0.28	0.10	0.88	130.49	2.48	0.29	0.07	0.09	0.54	122.69	0.83	0.66
	37	0.42	0.09	−0.34	130.49	3.94	0.14	0.29	0.09	0.68	122.69	0.02	0.99
	38	0.57	0.10	−1.28	130.49	4.53	0.10	0.33	0.09	−0.01	122.69	0.74	0.69
	39	0.19	0.09	−1.90	130.49	4.95	0.08	−0.16	0.09	−0.98	122.69	3.85	0.15
**Testlets**													
Pain		−0.12	0.03	2.45	114.43	3.28	0.19	−0.09	0.02	0.02	107.57	0.61	0.74
Movements		0.11	0.03	1.20	114.43	1.88	0.39	0.12	0.02	1.47	107.57	0.58	0.75
Feelings		0.04	0.03	0.27	114.43	1.23	0.54	0.04	0.03	1.78	107.57	0.41	0.82
Disease		−0.12	0.02	−0.36	114.43	0.26	0.88	−0.11	0.02	0.14	107.57	0.84	0.66
Treatments		0.00	0.02	−0.41	114.43	2.81	0.25	0.09	0.02	1.06	107.57	0.62	0.73
Self-help		−0.07	0.03	−2.60	114.43	4.33	0.11	−0.08	0.02	−0.29	107.57	1.64	0.44
Support		0.15	0.03	−0.55	114.43	3.18	0.20	0.04	0.03	−0.40	107.57	3.44	0.18

*Loc* location, *SE* standard error, *FR* fit residuals, *DF* degrees of freedom, *RA* rheumatoid arthritis, *SSc* systemic sclerosis, *χ* ^*2*^ chi square

* Bonferroni adjusted *p* value > 0.0013 for model fit (i.e. 0.05/39 tests) or *p* > 0.0071 for subscale model fit (i.e. 0.05/7 tests)

Despite the generally good individual item fit within each of the domains, when the scale is assessed as a singular 39-item construct, the model fit suggests significant deviation from the Rasch model (Table 4, analysis 1). Further investigation revealed the major cause of this misfit to be multiple significant residual correlations between items, therefore indicating local dependency. The pattern of the residual correlations suggested that the dependency clustered within each of the separate domains; therefore, the items were grouped within their respective domains and treated as domain-level items (or testlets). This approach corrected for the local dependency within the domains, and subsequent analyses (Table 4, analysis 2 for the Polish, UK and the pooled datasets) resulted into fit to the model and satisfied the strict test for unidimensionality. The internal consistency was above the value of 0.7, which is a required value for group use [36].

**Table 4:** Fit statistics indicating item fit, person fit and unidimensionality of the 7-domain scales in SSc and RA disease groups

Disease group	Country	Analyses	Item fit residual		Person fit residual		Chi-square interaction		PSI	Independent *t* tests (95 % CI)
			Mean	SD	Mean	SD	Value (*df*)	*p*		
SSc	Poland	Analysis 1	0.362	1.142	−0.411	2.312	104.562 (78)	0.024	0.947	
		Analysis 2	0.540	0.883	−0.329	1.233	8.132 (14)	0.882	0.855	0.046 (0.008, 0.083)
	UK	Analysis 1	0.319	1.524	−0.330	2.105	54.951 (39)	0.047	0.933	
		Analysis 2	0.391	0.723	−0.336	1.166	7.439 (7)	0.385	0.816	0.056 (0.018, 0.094)
	Pooled	Analysis 1	0.434	1.913	−0.441	2.302	226.122 (156)	<0.001	0.940	
		Analysis 2	0.567	0.598	−0.368	1.220	24.189 (28)	0.672	0.829	0.056 (0.029, 0.084)
		DIF-adjusted	0.520	0.559	−0.356	1.218	33.259 (32)	0.406	0.838	
RA	Poland	Analysis 1	0.142	1.826	−0.556	2.429	151.258 (78)	<0.001	0.959	
		Analysis 2	0.000	1.573	−0.428	1.213	16.953 (14)	0.259	0.894	0.069 (0.034, 0.105)
	UK	Analysis 1	0.288	1.868	−0.250	2.133	101.958 (39)	<0.001	0.932	
		Analysis 2	0.236	0.953	−3.955	1.290	6.392 (7)	0.495	0.857	0.069 (0.046, 0.143)
	Pooled	Analysis 1	0.041	2.637	−0.450	2.455	345.946 (117)	<0.001	0.954	
		Analysis 2	−0.003	1.646	−0.440	1.258	28.143 (21)	0.136	0.885	0.067 (0.041, 0.093)
		DIF-adjusted	0.163	1.151	−0.441	1.273	28.971 (33)	0.668	0.890	
Requirements for perfect fit			0	1	0	1		>0.05	>0.7	Lower-bound 95 % CI < 0.05

*Analysis 1* preliminary analysis with 39 items, *Analysis 2* analysis of subscales (testlets), *SD* standard deviation, *df* degrees of freedom, * *p* value > 0.5 for model fit, *PSI* person separation index

### Cross-cultural invariance

**Fig. 1:**
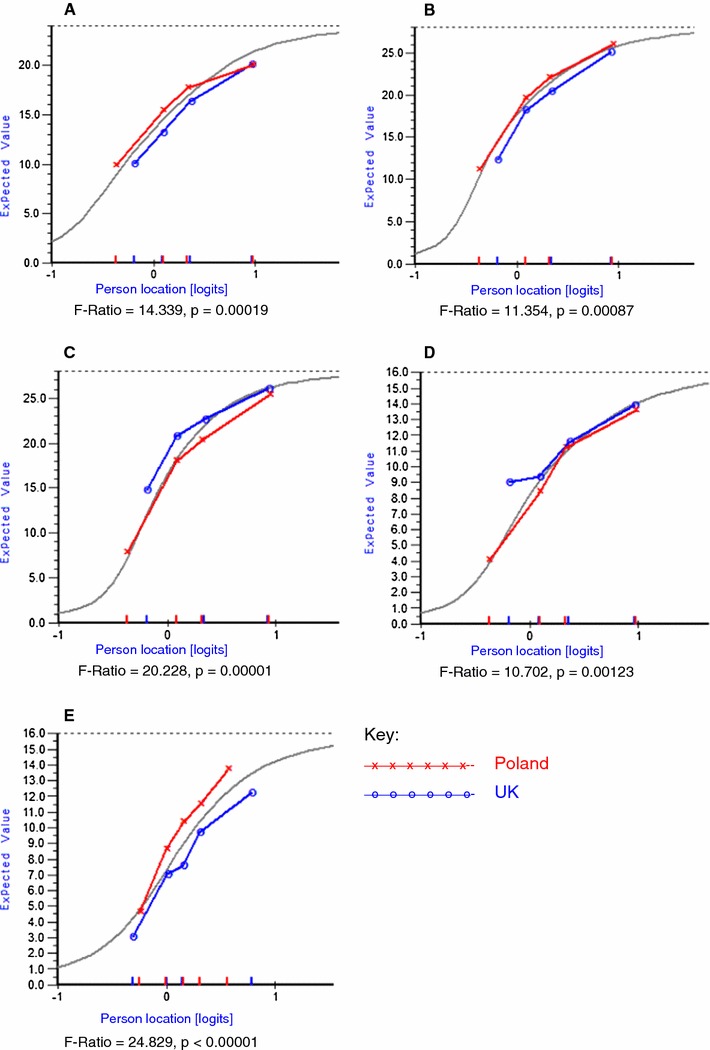
Cross-cultural DIF at domain (testlet) level in the RA and SSc disease groups. *RA* rheumatoid arthritis, *SSc* systemic sclerosis. **a** Pain (RA), **b** disease process (RA), **c** Treatments (RA), **d** Support (RA), **e** Support (SSc)

Polish Educational Needs Assessment Tool was invariant to age, gender, disease duration and education background. The pooled dataset for RA revealed a cross-cultural DIF where Polish patients were more likely to have higher scores on ‘pain’ and ‘disease process’ and lower scores on ‘treatments’ and ‘support’ than their UK counterparts. The SSc-pooled datasets revealed cross-cultural DIF in only one subscale—‘support’ where the Polish patients were more likely to have higher scores than their UK counterparts. The cross-cultural DIF patterns and their significance are presented in Fig. 1. This finding means that adjustment for the cross-cultural bias is required if the data from Poland are pooled or compared with the UK data.

The Pol-ENAT raw scores for each of the seven subscales (which are ordinal in nature) were Rasch-transformed to calibrate interval-level scales with which data can be transformed. The conversion charts for the Polish data (RA and SSc) are presented in the online supplementary tables S1 and S2. Separate DIF-adjusted tables for comparison between the UK and Polish data are presented in online supplementary tables S3 and S4. The instruction on how to use the conversion charts is given in the online supplementary material.

## Discussion

Patient education should be an integral part of the care of people with rheumatic conditions [6, 10]. Our research has provided a valid tool with which clinicians in Poland can assess the educational needs of patients with RA and SSc. This assessment is likely to lead to provision of targeted and patient-centred education. When used for clinical purposes, the Pol-ENAT does not need scoring as the clinician can easily tell the items that are rated by the patient as ‘extremely important’. However, when used as an outcome measure, then the Pol-ENAT has satisfied psychometric standards to enable meaningful summation of scores within each domain. The tool has been found to have sufficient cross-cultural validity to enable data pooling and comparisons between Poland and the UK.

Although it is generally accepted that simple translation of a questionnaire into another language is not appropriate, there is no consensus on the best method for cross-cultural adaptation [37]. The current adaptation methods either use a ‘forward–backward’ translation or a ‘forward only’ translation. In this research, we have used the Beaton’s method [21], which utilizes ‘forward–backward’ translation. Proponents of a ‘forward only’ approach [38, 39] suggest that back-translation would cast doubt over the abilities of the forward translators, producing unmanageable amount of text and little information of any value. In our experience, the ‘forward–backward’ process has been valuable as evidenced in the results. The expert committee meeting and a field test with patients ensured thoroughness in translation, and a conceptual equivalence between the English and the Polish version of the ENAT was achieved. It is likely that other methods utilizing a ‘forward–backward’ translation [22–24, 40, 41] would have achieved similar results.

This study has demonstrated that several items did not achieve a ‘linguistic equivalence’ or ‘idiomatic equivalence’, and more cultural-specific terms had to be used to ensure that they are understood by the target population (Table 2). Even when the adaptation process is successful to an extent of achieving a linguistic equivalence, this does not guarantee construct validity and reliability or ‘measurement equivalence’ [26–29]. For this reason, Kucukdeveci et al. [35] have suggested that a psychometric test of cross-cultural invariance should be incorporated into the standard adaptation process, so that problematic items may be reviewed in the translation process.

Subjecting the adapted Pol-ENAT to Rasch analysis ensured that the measurement properties (construct validity and reliability) of the ENAT were retained following the adaptation process. Calibration of the Pol-ENAT into an interval scale enables transforming the raw data into interval level for parametric analyses if required. While it is possible to have a valid and a reliable adapted instrument which works well in a given culture, when cross-cultural DIF is present, it means that the instrument works differently at least in some of the items (or subscales), which possess DIF [27]. Cross-cultural DIF may mean that the tools are valid for use only in their respective countries, but not for multinational data comparisons or data pooling. Alternatively, the tools may need to be adjusted for the identified cross-cultural DIF to enable cross-cultural data comparison(s). In this study, Rasch analysis has confirmed a level of the tools’ cross-cultural validity sufficient for use between Poland and the UK, and we have calibrated DIF-adjusted conversion tables for use where comparison of the Polish and the UK data is required. This approach has been used in the previous cross-cultural validation of the ENAT [18, 19] and other questionnaires [35, 42, 43], which are intended for multinational use.

This study has three main limitations. First, convenience sample was used, and it was obtained from only six centres, therefore not necessarily representative of the whole Polish population. This is unlikely however to affect the conclusions of this study since Polish language has no major variations across the country and sample size requirements for Rasch analysis were adequately met. Second, the ENAT is a self-completed questionnaire, and consequently, it does not reach the population of patients who cannot read and write. Third, although the ENAT has been validated in other European countries [19], the DIF-adjusted conversion table developed in this research applies only to comparison between Poland and UK data. Comparisons with other countries can only take place when cross-cultural DIF patterns between Poland and those countries have been established. Future research should look into the impact of the advances in information and communication technologies and how tools such as Pol-ENAT could go beyond just assessment of needs, but also link patients with available and credible resources. Different versions of the ENAT can be obtained from the University of Leeds by following this link http://medhealth.leeds.ac.uk/info/732/psychometric_laboratory/1493/scales.

## Conclusion

Using a standard process in cross-cultural adaptation, conceptual equivalence was achieved between the original (UK) ENAT and the adapted Pol-ENAT. Fit to the Rasch model confirmed that the construct validity and internal consistency of the ENAT have been preserved. The scales have been calibrated to ensure psychometric equivalence when undertaking multinational research. The Pol-ENAT can be used with confidence in assessing the educational needs of patients with RA and SSc in Poland.

## Electronic supplementary material

Below is the link to the electronic supplementary material.

Supplementary material 1 (DOC 202 kb)

Supplementary material 2 (DOC 78 kb)
