# Supplementary material for: Developing the Polish Educational Needs Assessment Tool (Pol-ENAT) in rheumatoid arthritis and systemic sclerosis: a cross-cultural validation study using Rasch analysis
Source: Qual Life Res. 2014 Sep 18;24(3):721–33. doi: 10.1007/s11136-014-0805-6 (PMC4349946; doi:10.1007/s11136-014-0805-6)
Supplement: Supplementary file 1 — Supplementary material 1 (DOC 202 kb) [file 11136_2014_805_MOESM1_ESM.doc]

**Online supplementary table S1: Conversion of raw domain scores into Rasch-transformed domain scores for use with Polish RA data**

| Raw scores | Transformed domain scores | | | | | | |
| --- | --- | --- | --- | --- | --- | --- | --- |
|  | Pain | Movement | Feelings | Arthritis | Treatments | Self-Help | Support |
| 0 | 0.0 | 0.0 | 0.0 | 0.0 | 0.0 | 0.0 | 0.0 |
| 1 | 2.1 | 1.4 | 1.1 | 1.1 | 1.3 | 2.1 | 0.8 |
| 2 | 3.5 | 2.4 | 2.0 | 1.9 | 2.3 | 3.5 | 1.5 |
| 3 | 4.4 | 3.1 | 2.7 | 2.4 | 3.0 | 4.4 | 2.1 |
| 4 | 5.2 | 3.6 | 3.4 | 2.9 | 3.6 | 5.0 | 2.6 |
| 5 | 5.9 | 4.1 | 4.1 | 3.3 | 4.1 | 5.6 | 3.1 |
| 6 | 6.4 | 4.6 | 4.8 | 3.6 | 4.6 | 6.1 | 3.6 |
| 7 | 7.0 | 5.1 | 5.7 | 3.9 | 5.1 | 6.5 | 4.1 |
| 8 | 7.5 | 5.6 | 6.8 | 4.2 | 5.5 | 6.9 | 4.7 |
| 9 | 8.1 | 6.2 | 8.2 | 4.6 | 6.0 | 7.3 | 5.4 |
| 10 | 8.7 | 6.8 | 9.4 | 4.9 | 6.4 | 7.8 | 6.4 |
| 11 | 9.3 | 7.5 | 10.5 | 5.2 | 6.8 | 8.2 | 7.6 |
| 12 | 9.9 | 8.2 | 11.4 | 5.6 | 7.4 | 8.6 | 8.9 |
| 13 | 10.5 | 9.1 | 12.3 | 6.0 | 7.9 | 9.1 | 10.1 |
| 14 | 11.2 | 10.1 | 13.4 | 6.4 | 8.5 | 9.6 | 11.6 |
| 15 | 12.0 | 11.2 | 14.6 | 6.9 | 9.2 | 10.1 | 13.5 |
| 16 | 12.7 | 12.4 | 16.0 | 7.4 | 10.1 | 10.8 | 16.0 |
| 17 | 13.6 | 13.7 |  | 8.2 | 11.0 | 11.6 |  |
| 18 | 14.4 | 15.2 |  | 9.1 | 12.0 | 12.5 |  |
| 19 | 15.3 | 17.2 |  | 10.1 | 13.0 | 13.5 |  |
| 20 | 16.3 | 20.0 |  | 11.3 | 14.0 | 14.7 |  |
| 21 | 17.5 |  |  | 12.5 | 15.0 | 16.1 |  |
| 22 | 18.9 |  |  | 13.8 | 16.1 | 17.8 |  |
| 23 | 21.0 |  |  | 15.1 | 17.2 | 20.4 |  |
| 24 | 24.0 |  |  | 16.6 | 18.4 | 24.0 |  |
| 25 |  |  |  | 18.4 | 19.8 |  |  |
| 26 |  |  |  | 20.5 | 21.6 |  |  |
| 27 |  |  |  | 23.6 | 24.2 |  |  |
| 28 |  |  |  | 28.0 | 28.0 |  |  |

**Online supplementary table S2: Conversion of raw domain scores into Rasch-transformed domain scores for use with Polish SSc data**

| **Raw scores** | **Rasch-transformed scores** | | | | | | |
| --- | --- | --- | --- | --- | --- | --- | --- |
|  | Pain | Movement | Feelings | Arthritis | Treatments | Self-Help | Support |
| 0 | 0.0 | 0.0 | 0.0 | 0.0 | 0.0 | 0.0 | 0.0 |
| 1 | 1.2 | 1.7 | 1.6 | 3.0 | 0.6 | 0.7 | 1.4 |
| 2 | 2.2 | 2.9 | 2.8 | 4.8 | 1.2 | 1.3 | 2.4 |
| 3 | 2.9 | 3.7 | 3.7 | 6.0 | 1.7 | 2.0 | 3.1 |
| 4 | 3.4 | 4.5 | 4.5 | 6.8 | 2.0 | 2.6 | 3.7 |
| 5 | 4.0 | 5.1 | 5.2 | 7.5 | 2.4 | 3.1 | 4.2 |
| 6 | 4.4 | 5.9 | 5.8 | 8.0 | 2.7 | 3.5 | 4.7 |
| 7 | 5.0 | 6.6 | 6.4 | 8.5 | 3.0 | 4.1 | 5.2 |
| 8 | 5.4 | 7.2 | 7.1 | 8.9 | 3.3 | 4.5 | 5.7 |
| 9 | 6.0 | 8.0 | 7.7 | 9.4 | 3.6 | 5.1 | 6.2 |
| 10 | 6.6 | 8.8 | 8.4 | 9.7 | 3.8 | 5.7 | 6.8 |
| 11 | 7.2 | 9.7 | 9.2 | 10.1 | 4.0 | 6.3 | 7.5 |
| 12 | 8.0 | 10.6 | 10.0 | 10.5 | 4.4 | 7.1 | 8.4 |
| 13 | 9.0 | 11.5 | 11.0 | 10.9 | 4.6 | 8.3 | 9.5 |
| 14 | 10.1 | 12.4 | 12.2 | 11.3 | 4.9 | 10.1 | 10.9 |
| 15 | 11.3 | 13.3 | 13.8 | 11.6 | 5.2 | 12.0 | 13.0 |
| 16 | 12.5 | 14.3 | 16.0 | 12.0 | 5.6 | 13.4 | 16.0 |
| 17 | 13.5 | 15.3 |  | 12.5 | 6.0 | 14.6 |  |
| 18 | 14.5 | 16.4 |  | 12.9 | 6.5 | 15.6 |  |
| 19 | 15.6 | 17.9 |  | 13.4 | 7.3 | 16.7 |  |
| 20 | 16.7 | 20.0 |  | 14.0 | 8.2 | 17.7 |  |
| 21 | 17.9 |  |  | 14.6 | 9.5 | 18.9 |  |
| 22 | 19.3 |  |  | 15.4 | 11.0 | 20.3 |  |
| 23 | 21.3 |  |  | 16.3 | 12.6 | 22.0 |  |
| 24 | 24.0 |  |  | 17.3 | 14.3 | 24.0 |  |
| 25 |  |  |  | 18.7 | 16.4 |  |  |
| 26 |  |  |  | 20.6 | 18.9 |  |  |
| 27 |  |  |  | 23.5 | 22.6 |  |  |
| 28 |  |  |  | 28.0 | 28.0 |  |  |

**Online supplementary table S3: DIF-adjusted conversion of raw domain scores into Rasch-transformed domain scores – for comparing Polish and UK SSc data**

| **Raw scores** | **Rasch-transformed scores** | | | | | | | |
| --- | --- | --- | --- | --- | --- | --- | --- | --- |
|  | Pain | Movement | Feelings | Arthritis | Treatments | Self-Help | Support  UK | Support Poland |
| 0 | 0.0 | 0.0 | 0.0 | 0.0 | 0.0 | 0.0 | 0.0 | 0.0 |
| 1 | 1.2 | 1.8 | 1.8 | 2.7 | 0.9 | 5.1 | 1.9 | 1.5 |
| 2 | 2.2 | 3.2 | 3.2 | 4.4 | 1.6 | -0.8 | 3.3 | 2.5 |
| 3 | 2.9 | 4.2 | 4.1 | 5.6 | 2.2 | 5.9 | 4.2 | 3.2 |
| 4 | 3.5 | 5.0 | 5.0 | 6.5 | 2.8 | 6.5 | 5.0 | 3.8 |
| 5 | 4.0 | 5.9 | 5.7 | 7.2 | 3.3 | 6.8 | 5.7 | 4.4 |
| 6 | 4.5 | 6.6 | 6.4 | 7.8 | 3.7 | 7.1 | 6.4 | 4.8 |
| 7 | 5.1 | 7.3 | 7.0 | 8.4 | 4.0 | 7.5 | 7.1 | 5.3 |
| 8 | 5.5 | 8.1 | 7.6 | 8.9 | 4.4 | 7.9 | 7.7 | 5.8 |
| 9 | 6.1 | 8.8 | 8.3 | 9.5 | 4.8 | 8.2 | 8.4 | 6.4 |
| 10 | 6.7 | 9.6 | 8.9 | 9.9 | 5.1 | 8.6 | 9.1 | 7.0 |
| 11 | 7.4 | 10.4 | 9.6 | 10.4 | 5.4 | 9.1 | 9.8 | 7.7 |
| 12 | 8.2 | 11.2 | 10.4 | 10.9 | 5.8 | 9.7 | 10.6 | 8.5 |
| 13 | 9.3 | 12.0 | 11.2 | 11.3 | 6.2 | 10.4 | 11.4 | 9.5 |
| 14 | 10.6 | 12.7 | 12.3 | 11.8 | 6.6 | 11.6 | 12.4 | 10.9 |
| 15 | 11.8 | 13.6 | 13.9 | 12.3 | 7.0 | 13.4 | 13.9 | 13.0 |
| 16 | 13.0 | 14.4 | 16.0 | 12.8 | 7.5 | 14.7 | 16.0 | 16.0 |
| 17 | 14.1 | 15.4 |  | 13.3 | 8.1 | 15.8 |  |  |
| 18 | 15.1 | 16.5 |  | 13.9 | 8.7 | 16.7 |  |  |
| 19 | 16.1 | 17.9 |  | 14.5 | 9.5 | 17.6 |  |  |
| 20 | 17.1 | 20.0 |  | 15.1 | 10.5 | 18.5 |  |  |
| 21 | 18.2 |  |  | 15.8 | 11.6 | 19.5 |  |  |
| 22 | 19.6 |  |  | 16.6 | 12.8 | 20.7 |  |  |
| 23 | 21.5 |  |  | 17.5 | 14.2 | 22.2 |  |  |
| 24 | 24.0 |  |  | 18.5 | 15.7 | 24.0 |  |  |
| 25 |  |  |  | 19.8 | 17.4 |  |  |  |
| 26 |  |  |  | 21.5 | 19.7 |  |  |  |
| 27 |  |  |  | 24.0 | 23.0 |  |  |  |
| 28 |  |  |  | 28.0 | 28.0 |  |  |  |

**Online supplementary table S4: DIF-adjusted conversion of raw domain scores into Rasch-transformed domain scores– for comparing Polish and UK RA data**

| **Raw scores** | **Rasch-transformed scores** | | | | | | | | | | |
| --- | --- | --- | --- | --- | --- | --- | --- | --- | --- | --- | --- |
|  | Pain  UK | Poland Pain | Movement  Both | Feelings  Both | Arthritis UK | Arthritis Poland | Treatments UK | Treatments Poland | Self-Help  Both | Support UK | Support Poland |
| 0 | 0.0 | 0.0 | 0.0 | 0.0 | 0.0 | 0.0 | 0.0 | 0.0 | 0.0 | 0.0 | 0.0 |
| 1 | 1.0 | 2.2 | 1.8 | 1.2 | 0.7 | 0.9 | 1.1 | 1.3 | 2.0 | 0.5 | 0.8 |
| 2 | 1.7 | 3.6 | 3.1 | 2.2 | 1.2 | 1.6 | 1.9 | 2.3 | 3.3 | 0.9 | 1.5 |
| 3 | 2.2 | 4.6 | 4.0 | 2.8 | 1.7 | 2.2 | 2.5 | 3.1 | 4.1 | 1.2 | 2.1 |
| 4 | 2.7 | 5.3 | 4.7 | 3.5 | 2.1 | 2.7 | 3.0 | 3.7 | 4.7 | 1.6 | 2.6 |
| 5 | 3.1 | 6.0 | 5.4 | 4.1 | 2.5 | 3.0 | 3.4 | 4.2 | 5.3 | 1.9 | 3.1 |
| 6 | 3.5 | 6.6 | 6.0 | 4.7 | 2.8 | 3.4 | 3.8 | 4.7 | 5.7 | 2.3 | 3.6 |
| 7 | 4.0 | 7.2 | 6.7 | 5.4 | 3.2 | 3.7 | 4.0 | 5.1 | 6.2 | 2.6 | 4.1 |
| 8 | 4.4 | 7.7 | 7.3 | 6.2 | 3.5 | 4.1 | 4.4 | 5.6 | 6.6 | 3.0 | 4.7 |
| 9 | 4.9 | 8.3 | 8.0 | 7.1 | 3.8 | 4.4 | 4.7 | 6.1 | 6.9 | 3.5 | 5.4 |
| 10 | 5.4 | 8.8 | 8.7 | 8.2 | 4.2 | 4.7 | 5.1 | 6.5 | 7.4 | 4.2 | 6.4 |
| 11 | 6.1 | 9.4 | 9.4 | 9.2 | 4.5 | 5.1 | 5.4 | 7.0 | 7.8 | 6.0 | 7.6 |
| 12 | 7.1 | 10.0 | 10.2 | 10.2 | 4.9 | 5.4 | 5.7 | 7.5 | 8.2 | 8.0 | 8.9 |
| 13 | 8.3 | 10.7 | 11.0 | 11.2 | 5.3 | 5.8 | 6.1 | 8.0 | 8.7 | 9.5 | 10.2 |
| 14 | 9.8 | 11.4 | 11.8 | 12.4 | 5.8 | 6.2 | 6.5 | 8.6 | 9.2 | 11.2 | 11.6 |
| 15 | 11.1 | 12.1 | 12.6 | 13.9 | 6.5 | 6.7 | 7.0 | 9.3 | 9.9 | 13.3 | 13.5 |
| 16 | 12.4 | 12.8 | 13.5 | 16.0 | 7.4 | 7.3 | 7.5 | 10.1 | 10.6 | 16.0 | 16.0 |
| 17 | 13.6 | 13.6 | 14.6 |  | 8.5 | 8.0 | 8.2 | 11.0 | 11.4 |  |  |
| 18 | 14.8 | 14.5 | 15.8 |  | 9.8 | 8.9 | 9.0 | 12.0 | 12.3 |  |  |
| 19 | 15.8 | 15.4 | 17.5 |  | 11.1 | 10.0 | 10.0 | 13.0 | 13.4 |  |  |
| 20 | 17.0 | 16.4 | 20.0 |  | 12.4 | 11.1 | 11.1 | 14.0 | 14.7 |  |  |
| 21 | 18.1 | 17.5 |  |  | 13.6 | 12.3 | 12.3 | 15.0 | 16.1 |  |  |
| 22 | 19.5 | 19.0 |  |  | 14.9 | 13.6 | 13.5 | 16.1 | 17.9 |  |  |
| 23 | 21.4 | 21.0 |  |  | 16.3 | 15.0 | 14.9 | 17.2 | 20.4 |  |  |
| 24 | 24.0 | 24.0 |  |  | 17.7 | 16.5 | 16.4 | 18.4 | 24.0 |  |  |
| 25 |  |  |  |  | 19.3 | 18.3 | 18.2 | 19.8 |  |  |  |
| 26 |  |  |  |  | 21.2 | 20.4 | 20.3 | 21.6 |  |  |  |
| 27 |  |  |  |  | 24.0 | 23.5 | 23.5 | 24.2 |  |  |  |
| 28 |  |  |  |  | 28.0 | 28.0 | 28.0 | 28.0 |  |  |  |
