# Supplementary material for: Developing the Polish Educational Needs Assessment Tool (Pol-ENAT) in rheumatoid arthritis and systemic sclerosis: a cross-cultural validation study using Rasch analysis
Source: Qual Life Res. 2014 Sep 18;24(3):721–33. doi: 10.1007/s11136-014-0805-6 (PMC4349946; doi:10.1007/s11136-014-0805-6)
Supplement: Supplementary file 2 — Supplementary material 2 (DOC 78 kb) [file 11136_2014_805_MOESM2_ESM.doc]

# Supplementary material:

**How to use and score the Educational Needs Assessment Tool (The ENAT)**

**Background information**

The Educational Needs Assessment Tool is a self-administered questionnaire containing 39 statements, which measure the educational needs of patients with rheumatic diseases. The statements are grouped into 7 domains that measure specific aspects of “educational needs”. These are:

1. Managing pain
2. Movement
3. Feelings
4. Disease process
5. Treatment form health professionals
6. Self-help measures
7. Support from others

**Uses of the ENAT**

The ENAT can be used by clinicians in wards or clinics to assess what are the most important educational / informational needs from the patient’s point of view. This information, along with the clinicians’ perception of what the patient needs to know, will allow the provision of timely and meaningful education tailored to the needs of each individual patient.

The ENAT can also be used to assess the educational needs of groups of patients (or group comparisons).

**How the ENAT is completed**

The ENAT is designed to be completed by the patient unaided. This gives objective information which is free from bias. The first page gives information about personal characteristics and an overall indication of the patient’s educational needs. This page is not an integral part of the ENAT and it is up to the practitioner to decide whether this information is required. For instance it may be of use for research purposes, but not for clinical practice.

The actual ENAT items start on page 2. They comprise 5-point Likert scales with the following descriptives: “Not at all important”, “A little important”, “Fairly important”, “Very important” and “Extremely important”.

From this point onwards, patients should put a tick “✓” in the box that corresponds with the level of importance they place on each statement. Only one box should be ticked for each statement.

**How is the ENAT scored?**

The ENAT can be used as a routine clinical checklist or as a research (or an audit) tool.

1. **ENAT used as a clinical checklist**

The clinician may want to know which item is most important for the patient at a particular time which will allow her/him to focus on this item during their consultation. In this case, the perceived priority needs can be determined by looking at the completed ENAT without the need for scoring (Example 1).

Example 1: This item relates to your feelings:

| **How important is it for you NOW to know more about the following:** | Not at all important | A little important | Fairly  Important | Very  Important | Extremely important |
| --- | --- | --- | --- | --- | --- |
| Ways to deal with stress | ✓ |  |  |  |  |
| Ways to deal with moods or depression |  | ✓ |  |  |  |
| Why I am feeling tired |  |  |  |  | ✓ |
| Why I am feeling down or depressed |  |  |  | ✓ |  |

1. **ENAT used as a survey or audit tool**

For use in audit or in research, the ENAT needs to be coded and scored using the following steps:

1. The ENAT Likert scales descriptives should be number-coded thus:

Not at all important = 0

A little important = 1

Fairly important = 2

Very important = 3

Extremely important = 4

See example 2 below:

Example 2: This item relates to your feelings:

| **How important is it for you NOW to know more about the following:** | Not at all important  **(0)** | A little important  **(1)** | Fairly  Important  **(2)** | Very  Important  **(3)** | Extremely important  **(4)** |
| --- | --- | --- | --- | --- | --- |
| Ways to deal with stress | ✓ |  |  |  |  |
| Ways to deal with moods or depression |  | ✓ |  |  |  |
| Why I am feeling tired |  |  |  |  | ✓ |
| Why I am feeling down or depressed |  |  |  | ✓ |  |

1. Following the coding, the scores for each statement are then added up to provide the **raw domain score**. In the example above, the domain score = 8 (the domain score for feelings should range between 0 – 16).
2. The raw domain scores obtained in (ii) can be transformed into linear scale to enable their use in parametric analyses. This is done by using the conversion tables provided for specific disease cohort. The table below is specific for use in Polish population with rheumatoid arthritis. For example, for feelings, the raw domain score of 8, corresponds to **transformed domain score** of 6.8 (See the conversion table below).
3. Adding up all the transformed domain scores gives the **total ENAT score**, which is an estimate of the patient’s educational, needs (range = 0 -156).

**Conversion of raw Domain scores into Rasch-transformed domain scores (for RA)**

| Raw scores | Transformed domain scores | | | | | | |
| --- | --- | --- | --- | --- | --- | --- | --- |
|  | Pain | Movement | Feelings | Arthritis | Treatments | Self-Help | Support |
| 0 | 0.0 | 0.0 | 0.0 | 0.0 | 0.0 | 0.0 | 0.0 |
| 1 | 2.1 | 1.4 | 1.1 | 1.1 | 1.3 | 2.1 | 0.8 |
| 2 | 3.5 | 2.4 | 2.0 | 1.9 | 2.3 | 3.5 | 1.5 |
| 3 | 4.4 | 3.1 | 2.7 | 2.4 | 3.0 | 4.4 | 2.1 |
| 4 | 5.2 | 3.6 | 3.4 | 2.9 | 3.6 | 5.0 | 2.6 |
| 5 | 5.9 | 4.1 | 4.1 | 3.3 | 4.1 | 5.6 | 3.1 |
| 6 | 6.4 | 4.6 | 4.8 | 3.6 | 4.6 | 6.1 | 3.6 |
| 7 | 7.0 | 5.1 | 5.7 | 3.9 | 5.1 | 6.5 | 4.1 |
| 8 | 7.5 | 5.6 | 6.8 | 4.2 | 5.5 | 6.9 | 4.7 |
| 9 | 8.1 | 6.2 | 8.2 | 4.6 | 6.0 | 7.3 | 5.4 |
| 10 | 8.7 | 6.8 | 9.4 | 4.9 | 6.4 | 7.8 | 6.4 |
| 11 | 9.3 | 7.5 | 10.5 | 5.2 | 6.8 | 8.2 | 7.6 |
| 12 | 9.9 | 8.2 | 11.4 | 5.6 | 7.4 | 8.6 | 8.9 |
| 13 | 10.5 | 9.1 | 12.3 | 6.0 | 7.9 | 9.1 | 10.1 |
| 14 | 11.2 | 10.1 | 13.4 | 6.4 | 8.5 | 9.6 | 11.6 |
| 15 | 12.0 | 11.2 | 14.6 | 6.9 | 9.2 | 10.1 | 13.5 |
| 16 | 12.7 | 12.4 | 16.0 | 7.4 | 10.1 | 10.8 | 16.0 |
| 17 | 13.6 | 13.7 |  | 8.2 | 11.0 | 11.6 |  |
| 18 | 14.4 | 15.2 |  | 9.1 | 12.0 | 12.5 |  |
| 19 | 15.3 | 17.2 |  | 10.1 | 13.0 | 13.5 |  |
| 20 | 16.3 | 20.0 |  | 11.3 | 14.0 | 14.7 |  |
| 21 | 17.5 |  |  | 12.5 | 15.0 | 16.1 |  |
| 22 | 18.9 |  |  | 13.8 | 16.1 | 17.8 |  |
| 23 | 21.0 |  |  | 15.1 | 17.2 | 20.4 |  |
| 24 | 24.0 |  |  | 16.6 | 18.4 | 24.0 |  |
|  |  |  |  | 18.4 | 19.8 |  |  |
|  |  |  |  | 20.5 | 21.6 |  |  |
|  |  |  |  | 23.6 | 24.2 |  |  |
|  |  |  |  | 28.0 | 28.0 |  |  |
